# Three-photon tissue imaging using moxifloxacin

**DOI:** 10.1038/s41598-018-27371-8

**Published:** 2018-06-20

**Authors:** Seunghun Lee, Jun Ho Lee, Taejun Wang, Won Hyuk Jang, Yeoreum Yoon, Bumju Kim, Yong Woong Jun, Myoung Joon Kim, Ki Hean Kim

**Affiliations:** 1 Department of Mechanical Engineering, Pohang University of Science and Technology, 77 Cheongam-ro, Nam-gu, Pohang, Gyeongbuk 37673 Republic of Korea; 2 Division of Integrative Biosciences and Biotechnology, Pohang University of Science and Technology, 77 Cheongam-ro, Nam-gu, Pohang, Gyeongbuk 37673 Republic of Korea; 3 Department of Chemistry, Pohang University of Science and Technology, 77 Cheongam-ro, Nam-gu, Pohang, Gyeongbuk 37673 Republic of Korea; 4 Department of Ophthalmology, University of Ulsan College of Medicine, Asan Medical Center, 88 Olympic-ro 43-gil, Songpa-gu, Seoul, 05505 Republic of Korea

## Abstract

Moxifloxacin is an antibiotic used in clinics and has recently been used as a clinically compatible cell-labeling agent for two-photon (2P) imaging. Although 2P imaging with moxifloxacin labeling visualized cells inside tissues using enhanced fluorescence, the imaging depth was quite limited because of the relatively short excitation wavelength (<800 nm) used. In this study, the feasibility of three-photon (3P) excitation of moxifloxacin using a longer excitation wavelength and moxifloxacin-based 3P imaging were tested to increase the imaging depth. Moxifloxacin fluorescence via 3P excitation was detected at a >1000 nm excitation wavelength. After obtaining the excitation and emission spectra of moxifloxacin, moxifloxacin-based 3P imaging was applied to *ex vivo* mouse bladder and *ex vivo* mouse small intestine tissues and compared with moxifloxacin-based 2P imaging by switching the excitation wavelength of a Ti:sapphire oscillator between near 1030 and 780 nm. Both moxifloxacin-based 2P and 3P imaging visualized cellular structures in the tissues via moxifloxacin labeling, but the image contrast was better with 3P imaging than with 2P imaging at the same imaging depths. The imaging speed and imaging depth of moxifloxacin-based 3P imaging using a Ti:sapphire oscillator were limited by insufficient excitation power. Therefore, we constructed a new system for moxifloxacin-based 3P imaging using a high-energy Yb fiber laser at 1030 nm and used it for *in vivo* deep tissue imaging of a mouse small intestine. Moxifloxacin-based 3P imaging could be useful for clinical applications with enhanced imaging depth.

## Introduction

Two-photon microscopy (2PM) is a nonlinear fluorescence microscopy technique based on two-photon (2P) excitation of fluorophores^1^. 2PM has been used in various biological studies at the cellular level, including neurobiology^2^, immunology^3^, and cancer biology^4^. It has a relatively deep imaging depth and low phototoxicity compared to those of other three-dimensional (3D) fluorescence microscopy techniques such as confocal microscopy^5^. 2PM can image tissues without exogenous labeling by utilizing intrinsic tissue contrast such as autofluorescence and second harmonic generation (SHG) and has been used in both preclinical^6^ and clinical studies^7^. However, use of label-free 2PM in clinical studies was limited by low intrinsic signal levels and subsequent low imaging speeds. Recently, we demonstrated that moxifloxacin can be used as a cell-labeling agent for 2PM^8^. Moxifloxacin is an FDA-approved antibiotic used in the clinic to both treat and prevent bacterial infections. It has intrinsic fluorescence^9^ as well as good pharmacokinetic properties such as relatively high aqueous solubility and lipophilicity^10^ for tissue penetration. The 2P fluorescence of moxifloxacin was characterized and its application as a cell-labeling agent for 2PM was experimentally demonstrated^8^. Although 2P imaging of tissue using moxifloxacin as the cell-labeling agent was faster than label-free 2P imaging, its imaging depth was not large because of the relatively short excitation wavelength (<800 nm) used. The imaging depth of optical microscopy techniques, including 2PM, is typically limited by optical aberration and photon scattering. Adaptive optics-based methods, which correct optical aberration, have been developed to overcome the limitation in imaging depth. The effects of photon scattering decrease as the wavelength increases. Nonlinear methods that use a longer excitation wavelength than that used in 2PM, such as three-photon microscopy (3PM)^11^ and third-harmonic-generation microscopy (THGM)^12^, also have been developed to improve imaging depth. 3PM is a nonlinear fluorescence microscopy technique based on three-photon (3P) excitation of fluorophores. The longer excitation wavelength for a given fluorophore and the better 3D excitation confinement in scattering tissues allows 3PM to achieve deeper imaging than 2PM. However, 3P excitation is much less efficient than 2P excitation and requires excitation sources with a higher pulse energy. Chloroplast^13^, alveoli of mouse lung^14^, and RFP-labeled neurons^15^ have undergone 3P imaging.

In this study, we experimentally tested 3P excitation of moxifloxacin at an excitation wavelength of >1000 nm for deep tissue imaging with moxifloxacin labeling. Once moxifloxacin fluorescence was confirmed at this excitation wavelength range, its excitation and emission spectra were obtained. Moxifloxacin-based 3P imaging was performed on *ex vivo* mouse bladder and *ex vivo* small intestine tissues and compared with moxifloxacin-based 2P imaging by switching the excitation wavelength of a Ti:sapphire tunable oscillator. The performance of moxifloxacin-based 3P imaging was analyzed in terms of the image contrast. Lastly, we developed a new system for moxifloxacin-based 3P imaging that uses a high-power fiber laser at 1030 nm and demonstrated its ability to perform *in vivo* tissue imaging.

## Results

### Characterization of moxifloxacin fluorescence at >1000 nm excitation wavelength

**Figure 1:**
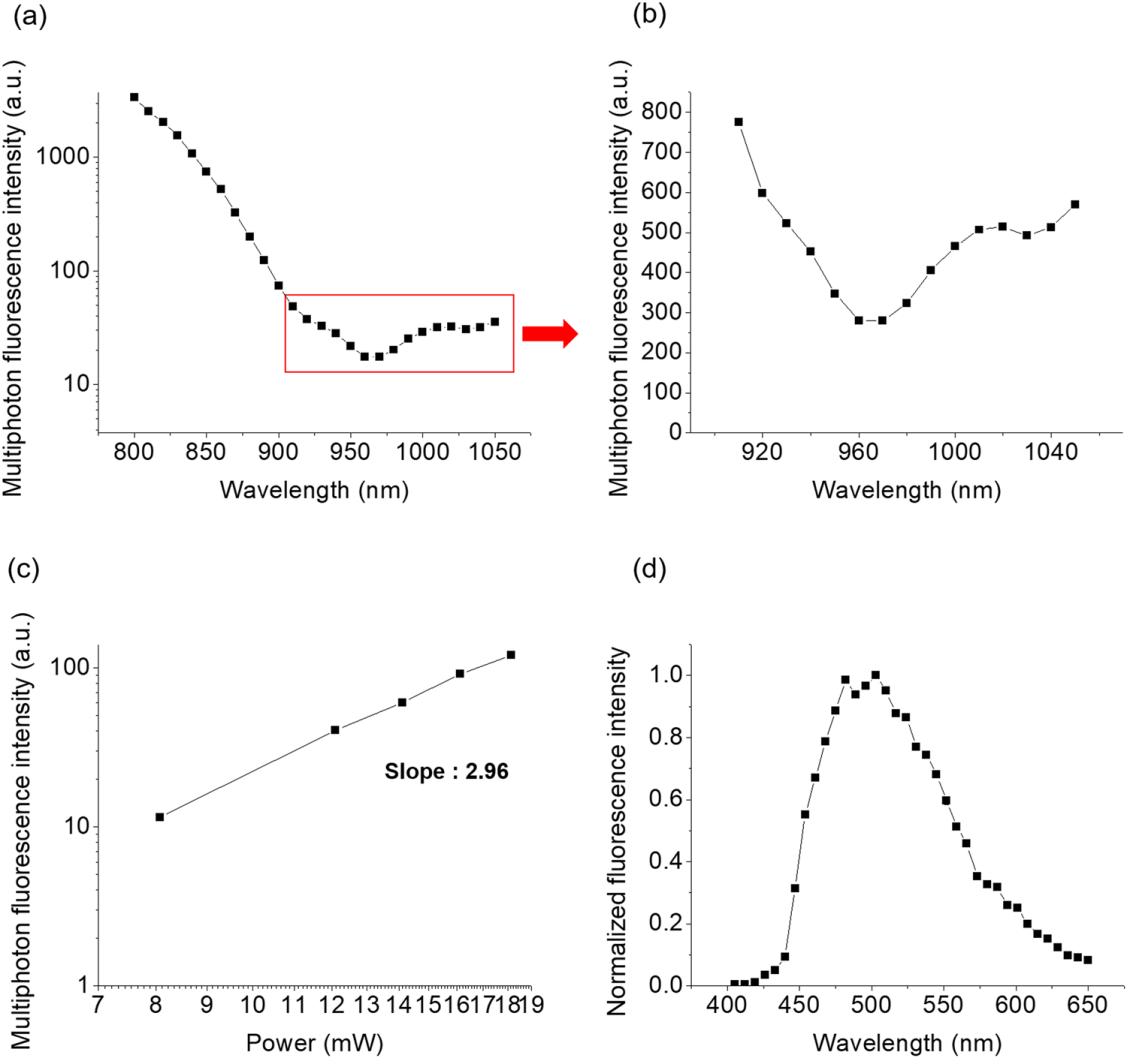
Fluorescence characteristics of moxifloxacin at a >1000 nm excitation wavelength. (**a**) Excitation spectrum of moxifloxacin from 800 to 1050 nm (*n* = 5). (**b**) Excitation spectrum of moxifloxacin from 900 to 1050 nm [enlargement of the boxed area in (**a**)]. (**c**) Log-log plot of fluorescence intensity as a function of excitation power (*n* = 10). (**d**) Emission spectrum of moxifloxacin at 1030 nm excitation wavelength (*n* = 5).

The excitation spectrum, the variation in fluorescence intensity with excitation power, and the emission spectrum of moxifloxacin are shown in Fig. 1a–b, c and d, respectively. The wavelength used for the excitation spectrum of moxifloxacin ranged from 800 to 1050 nm. The excitation spectrum decreased rapidly as the wavelength increased from 800 to 960 nm, because this wavelength range is far from the 2P excitation peak of moxifloxacin (<700 nm)^16^. For excitation wavelengths >960 nm, the excitation spectrum slowly increased as the wavelength increased (Fig. 1b). The increase in the moxifloxacin excitation spectrum at approximately 1000 nm is indicative of an excitation process different from that of 2P excitation, probably 3P excitation. The fluorescence intensity as a function of excitation power was measured to verify this probability (Fig. 1c). The excitation wavelength for this measurement was 1030 nm because there are commercially available light sources at this wavelength, e.g., Yb fiber femtosecond lasers. The fluorescence intensity of moxifloxacin increased with excitation power, and its slope was estimated to be 2.96 in log-log plot. Because the measured slope was close to 3, we confirmed that moxifloxacin excitation at 1030 nm was 3P excitation. The emission spectrum of moxifloxacin was also measured at an excitation wavelength of 1030 nm (Fig. 1d). It was quite broad, with its peak at approximately 510 nm. The 3P active cross section of moxifloxacin was 0.2 × 10^−83^ cm^6^/(s/photon)^2^, at the excitation wavelength of 1000 nm, approximately 0.8 times that of DAPI solution^17^ at the same concentration.

### Comparison of moxifloxacin-based 3P and 2P imaging of ***ex vivo*** mouse bladder

**Figure 2:**
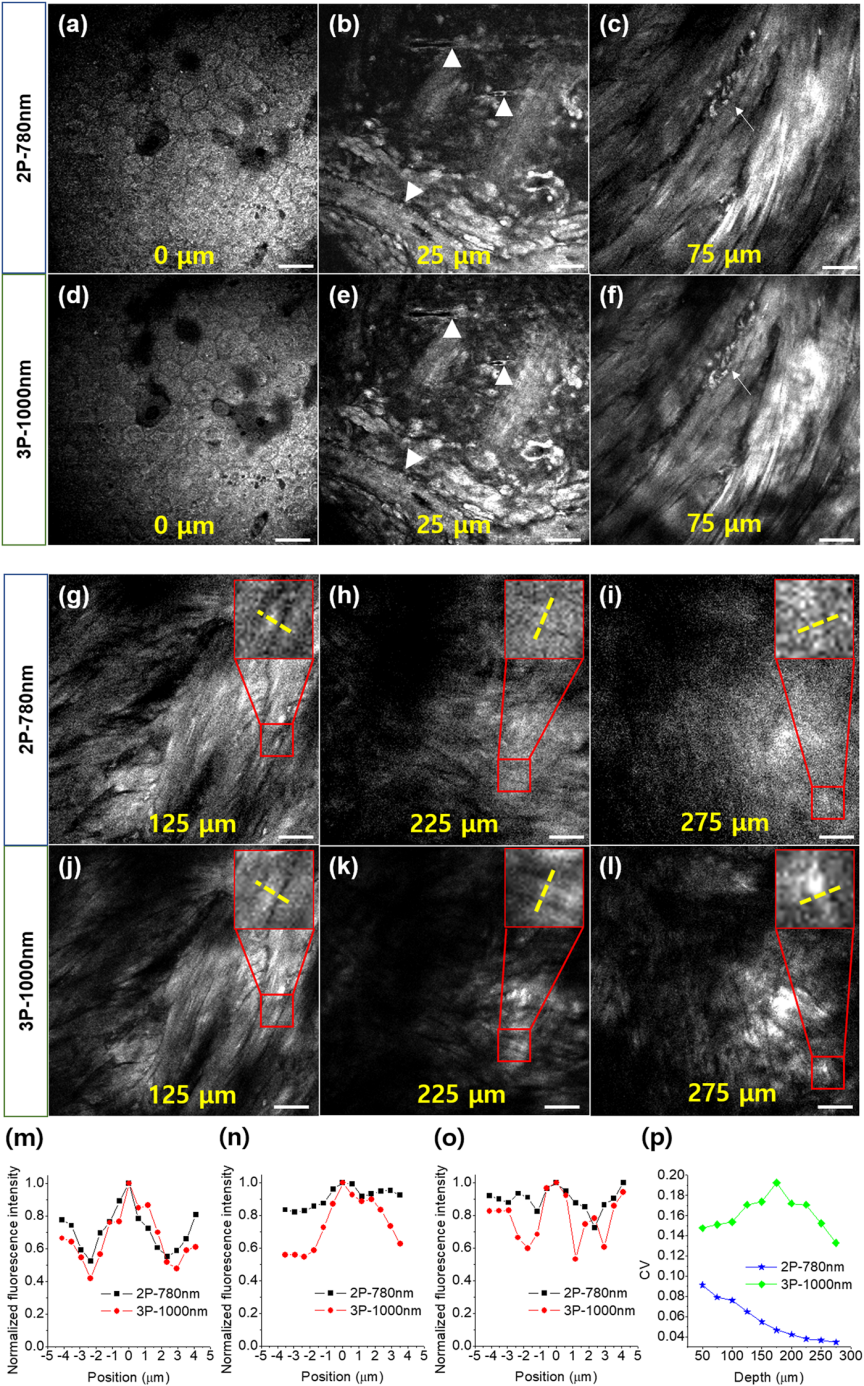
Moxifloxacin-based 2P and 3P images of an *ex vivo* mouse bladder at various depths from the luminal side. (**a**–**c**) 2P and (**d**–**f**) 3P images at depths of 0, 25, and 75 μm from the luminal surface. (**g**–**i**) 2P and (**j**–**l**) 3P images at depths of 125, 225, and 275 μm from the luminal surface. (**m**–**o**) Fluorescence intensity profile along the dashed yellow lines crossing the muscle cell in the insets of (**g**–**i**) and (**j**–**l**). (**p**) CV values at various depths in the muscle layer. Scale bar = 50 μm.

After characterizing moxifloxacin 3P fluorescence, moxifloxacin-based 3P imaging was tested in biological tissues and compared with moxifloxacin-based 2P imaging. First, moxifloxacin-based 2P and 3P imaging was conducted in *ex vivo* mouse bladder specimens by changing the excitation wavelength of the Ti:sapphire oscillator between 780 and 1000 nm, respectively. 2P and 3P images of the mouse bladder at several depths from the luminal side are presented in Fig. 2. For the 3P imaging, the excitation wavelength was 1000 nm instead of 1030 nm because SHG in the bladder was strong and moxifloxacin fluorescence needed to be distinguished from the SHG. The bladder is a muscular sac that stores urine generated by the kidney. Because various bladder disorders initially occur in the lumen, the luminal side underwent moxifloxacin-based imaging. Both 2P and 3P imaging with moxifloxacin labeling showed various cellular structures with depth, including hexagon-shaped umbrella cells in the uroepithelium, sparsely distributed cellular structures in the lamina propria, and a thick muscle layer below the lamina propria. The umbrella cells (Fig. 2a and d) and the cells in the lamina propria (Fig. 2b and e) were clearly visible via both moxifloxacin-based 2P and 3P imaging because of the cell labeling with moxifloxacin. Blood vessels (white arrowheads in Fig. 2b and e) in the lamina propria were visible via moxifloxacin labeling of vascular endothelial cells. Bundles of muscle cells and other small cells (white arrows in Fig. 2c and f) between the bundles were visible in the deep muscle layer.

Both the 2P and 3P images showed almost identical cellular structures via moxifloxacin labeling, and the anatomy captured by these imaging methods was consistent with histology^18^. Although both moxifloxacin-based 2P and 3P imaging visualized cellular structures clearly at relatively shallow depths, down to ~125 µm (Fig. 2g and j), the contrast of the moxifloxacin-based 2P images at deeper depths was less than that of the corresponding 3P images. At 225 µm from the surface, the moxifloxacin-based 2P image showed low contrast with a high background (Fig. 2h) and individual muscle cells were not well resolved. On the other hand, the moxifloxacin-based 3P image at the same depth showed distinct detailed muscle structures (Fig. 2k). At 275 µm from the surface, there was no contrast in the moxifloxacin-based 2P image (Fig. 2i). At the same depth, individual muscle cells could be seen in the moxifloxacin-based 3P image (Fig. 2l). Comparison of moxifloxacin-based 2P and 3P images of the mouse bladder specimen showed clearly that moxifloxacin-based 3P imaging can obtain deeper images by using a longer excitation wavelength and by better confinement of 3D excitation in the scattering tissue.

To analyze the image contrast, we plotted the intensity profiles (Fig. 2m–o) of the yellow dashed lines crossing the muscle cells in the moxifloxacin-based 2P and 3P images (insets in Fig. 2g–l). At 125 µm below the surface, the intensity profiles for both sets of images were high in the muscle cells and noticeably low in the regions between the muscle cells (Fig. 2m). At 225 µm below the surface, the intensity profile for the moxifloxacin-based 2P image showed a small peak in the muscle cells, with a slightly higher intensity than the surrounding area, while that for the moxifloxacin-based 3P image had a large peak in the muscle cells, with a noticeably stronger fluorescence intensity than the surrounding area (Fig. 2n). At 275 µm below the surface, the intensity profile of the moxifloxacin-based 2P image did not show distinguishable intensity variation between the muscle cells and surrounding area, while that of the moxifloxacin-based 3P image had noticeably high intensities in the muscle cells.

The image contrast of moxifloxacin-based 2P and 3P imaging was also analyzed quantitatively by calculating the coefficient of variation (CV), which is a measure of relative variability in the intensity image and calculated as the ratio of the standard deviation to the mean. Figure 2p shows the changes in the CV at several depths in muscle. The CV values for the moxifloxacin-based 2P images of the mouse bladder specimen monotonically decreased as the depth increased, which was consistent with the decrease in the image contrast. Moxifloxacin-based 3P images of the mouse bladder specimen had higher CV values than the corresponding 2P images at all depths in the muscle layer. However, the CV values of the 3P images decreased somewhat at depths >200 µm from the surface. At the maximum depth of 275 µm, the CV value of the 3P image was still quite high compared to that of the 2P image. This indicates that moxifloxacin-based 3P imaging still had good contrast at this depth.

### Comparison of moxifloxacin-based 3P and 2P imaging of ***ex vivo*** mouse small intestine

In addition to the mouse bladder specimen, moxifloxacin-based 3P imaging was conducted on *ex vivo* mouse small intestine specimens and compared with moxifloxacin-based 2P imaging. The small intestine, part of the gastrointestinal tract, absorbs nutrition from digested food and maintains gut microbiota. Intravital microscopy has been used in structural and functional studies of the small intestine but deep-tissue imaging methods would be more beneficial.

**Figure 3:**
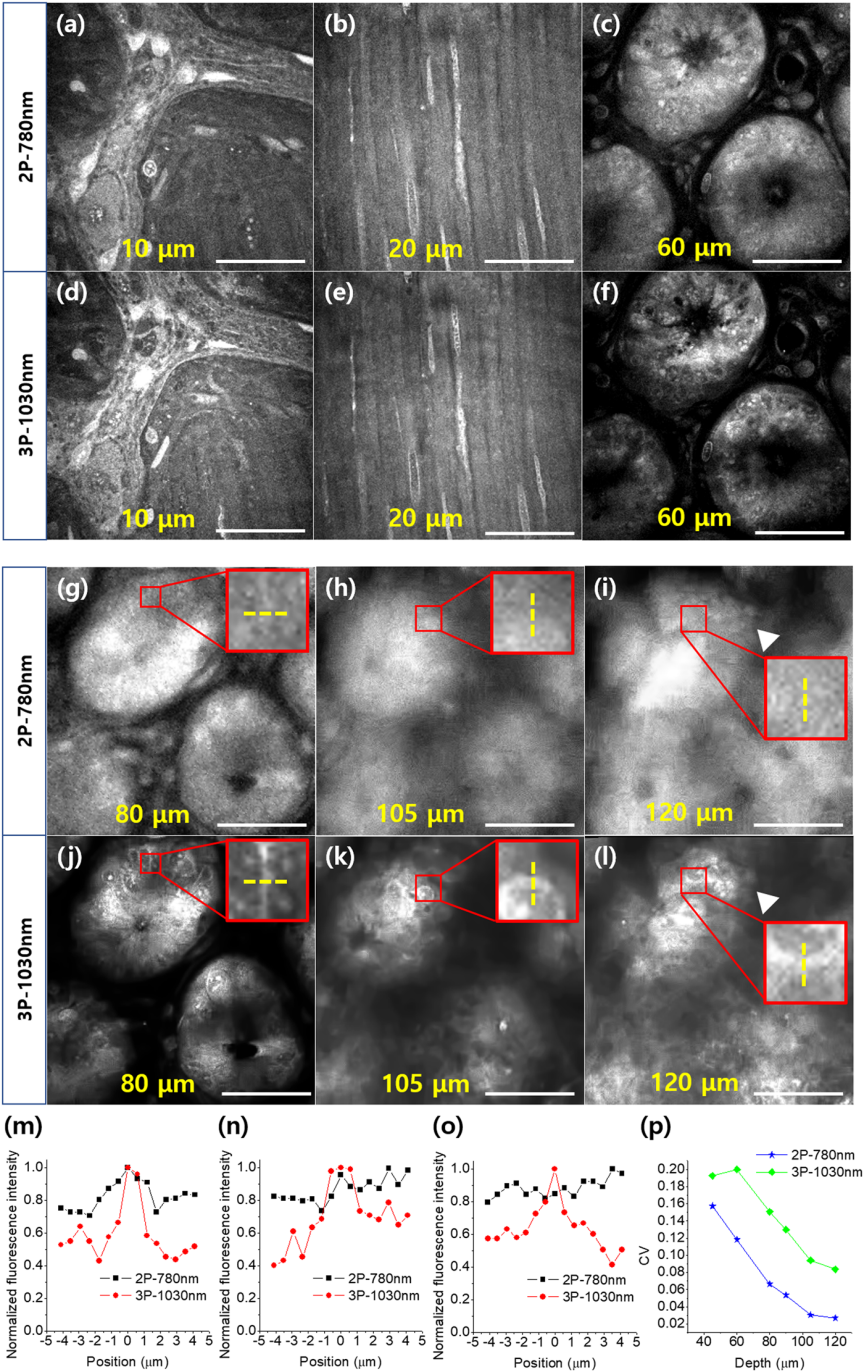
Moxifloxacin-based 2P and 3P images of an *ex vivo* mouse small intestine at various depths from the serosa. (**a**–**c**) 2P and (**d**–**f**) 3P images at depths of 10, 20, and 60 μm from the serosa. (**g**–**i**) 2P and (**j**–**l**) 3P images at depths of 80, 105, and 120 μm from the serosa. (**m**–**o**) Fluorescence intensity profile along the dashed yellow lines crossing the cell membrane in the insets of (**g**–**i**) and (**j**–**l**). (**p**) CV values at various depths in the crypt. Scale bar = 50 μm.

Moxifloxacin-based 2P and 3P imaging of the mouse small intestine specimen was conducted by switching the excitation wavelength of the Ti:sapphire oscillator between 780 and 1030 nm. Figure 3 shows moxifloxacin-based 2P and 3P images obtained at different depths from the serosa. Both moxifloxacin-based 2P and 3P imaging showed various cellular structures at different depths, including the myenteric plexus, muscle, and intestinal glands. The myenteric plexus, located between muscle layers, is part of the neuronal network in the gastrointestinal tract and is involved in the contractile and refractive motion used for digestion. Some cells in the myenteric plexus (Fig. 3a and d) and spindle-shaped cells in the muscle layer below the myenteric plexus (Fig. 3b and e) were clearly visible in both 2P and 3P images. Intestinal glands are depressed tubular glands in the lining of the epithelium that covers the lumen of the small and large intestines and contain Paneth cells and stem cells at the base and enterocytes and goblet cells in the lining. Images from both 2P and 3P imaging showed epithelial cells along the lining of the gland at the relatively shallow depth of 60 µm (Fig. 3c and f). However, at the depth of 80 µm, the epithelial cells in the moxifloxacin-based 2P image were barely distinguishable because of reduced contrast (Fig. 3g), and at larger depths, the individual epithelial cells could not be imaged at all (Fig. 3h). Only some cells in the lamina propria (white arrowhead in Fig. 3i) were visible down to 120 μm. On the other hand, image contrast was maintained in moxifloxacin-based 3P images and individual cells in deep regions of the glands could be seen (Fig. 3j–l).

To analyze the image contrast, we plotted the intensity profiles (Fig. 3m–o) of the yellow dashed lines crossing the membrane of the epithelial cells in the intestinal gland, seen in the insets of Fig. 3g–l. At 80 μm from the surface, the epithelial cell membrane showed a higher fluorescence signal intensity than the surrounding area in both moxifloxacin-based 2P and 3P images (Fig. 3m). At larger depths, there was no difference between the signal intensities of the cell membrane and the surrounding area in the moxifloxacin-based 2P images. However, moxifloxacin-based 3P images still had relatively high signal levels in the cell membrane (Fig. 3n and o).

We also calculated the CV values of the moxifloxacin-based 2P and 3P images of the mouse small intestine specimen; the change in the CV with depth is shown in Fig. 3p. The CV values of the moxifloxacin based 2P and 3P images rapidly decreased as the depth increased, probably because of the complex structure of the small intestine. The CV values of the 2P images were the lower than those of the corresponding 3P images at the same depths, which is consistent with the image contrast comparison. At the maximum depth of 120 µm from the surface, the CV value of the 2P image approached the minimum value of 0.025. However, the CV value of the corresponding 3P image was still high, indicating relatively good image contrast at 120 µm. This quantitative analysis of the image contrast showed that larger imaging depths could be reached in moxifloxacin-labeled tissues by using 3P excitation instead of 2P excitation.

### Moxifloxacin-based 3P imaging of ***in vivo*** mouse small intestine

Moxifloxacin-based 3P and 2P imaging techniques used on *ex vivo* biological tissues were compared by switching the excitation wavelength. Moxifloxacin-based 3P imaging of *in vivo* biological tissues was difficult with current multiphoton microscopy systems because the imaging takes a long time, especially at large depths (655 s/frame at a depth of 120 μm from the serosa surface of the mouse small intestine). The long imaging time is due to the energy limitation of the Ti:sapphire oscillators at a >1000 nm excitation wavelength. The excitation energy needed for moxifloxacin-based 3P imaging is approximately a few tenths of a nanojoule per pulse at large depths; however, the maximum excitation energy from a current Ti:sapphire oscillators are approximately 0.5 nJ at a >1000 nm excitation wavelength. Because the maximum excitation energy was used for moxifloxacin-based 3P imaging at the tissue surface, there was no extra energy to compensate for the loss of excitation energy as the depth increased. Therefore, the very long acquisition time was required to obtain a sufficient fluorescence signal at large depths, making *in vivo* moxifloxacin-based 3P imaging difficult. For *in vivo* moxifloxacin-based 3P imaging, we developed another 3P imaging system that uses a high-pulse-energy Yb fiber laser as the excitation source. This Yb fiber laser had a pulse energy of 10 µJ at 1034 nm and a pulse repetition rate of 102.9 kHz. It performed deep tissue imaging by compensating for the energy loss with increasing depth, although it had a very low pulse repetition rate for 3P imaging compared to that of the Ti:sapphire oscillator running at 80 MHz. The image acquisition rate was 66 s/frame, approximately 20 times slower than Ti:sapphire oscillator-based 3P imaging at the tissue surface due to the low repetition rate. However, the same acquisition rate could be maintained for all imaging depths and was much faster than that of Ti:sapphire oscillator-based 3P imaging at the large depths.

**Figure 4:**
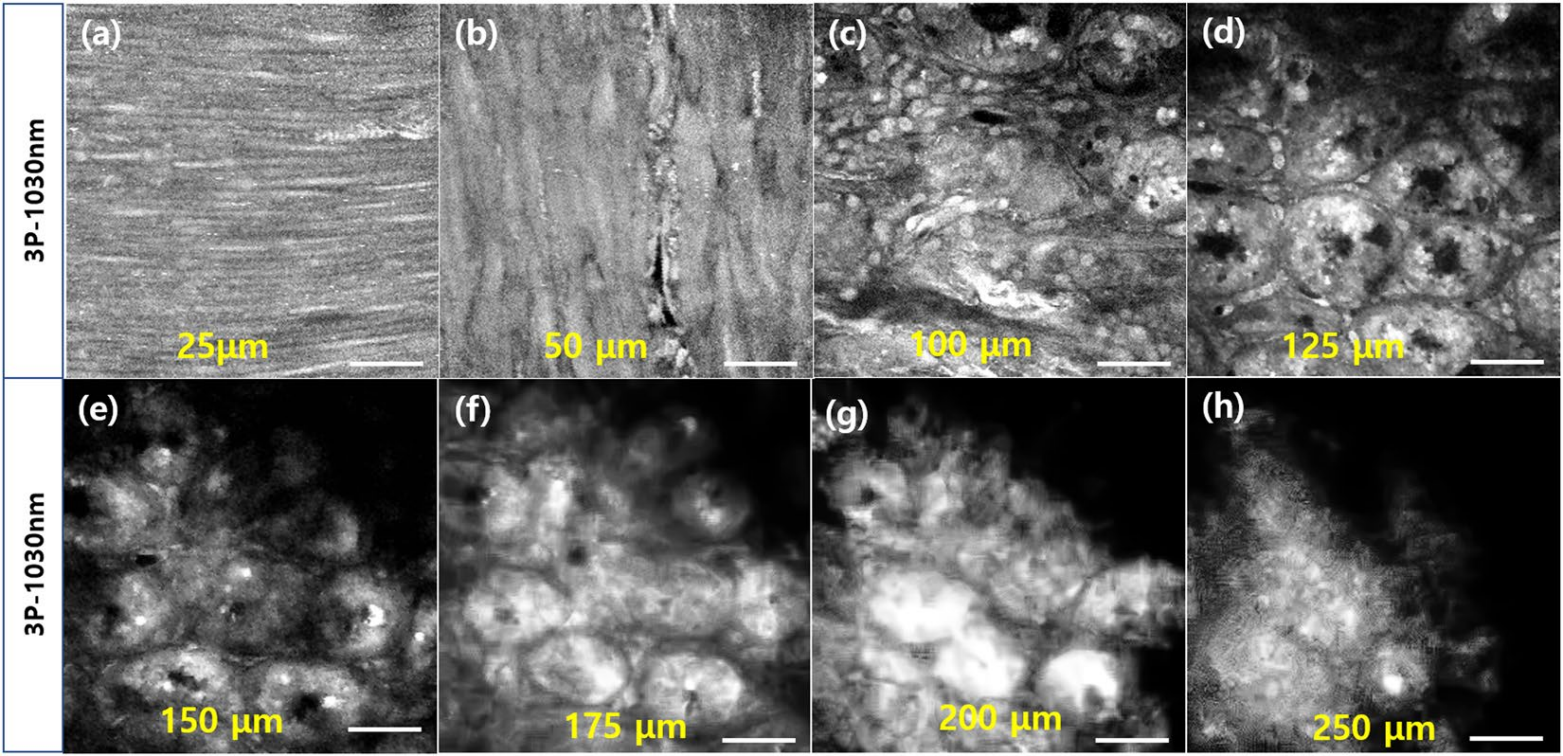
Moxifloxacin-based 3P images of an *in vivo* mouse small intestine at (**a**–**d**) depths of 25, 50, 100, and 125 μm from the serosa and (**e**–**h**) depths of 150, 175, 200, and 250 μm from the serosa. Scale bar = 50 μm.

*In vivo* imaging of the mouse small intestine in a gas-anesthetized mouse was conducted with the use of a small intestine holder; the details are discussed in the Methods section. Figure 4 presents moxifloxacin-based 3P images of the *in vivo* mouse small intestine at several depths from the surface of the serosa. *In vivo* moxifloxacin-based 3P imaging visualized the cellular structures of the small intestine, similar to the images acquired in the *ex vivo* study. Muscle layers oriented in alternating directions were visible at the shallow depths of 25 and 50 µm (Fig. 4a and b), and epithelial cells in tubular intestinal glands and surrounding cells in the lamina propria were visible at larger depths (Fig. 4c and d). Cells with bright fluorescent signals were imaged at a depth of 150 μm (Fig. 4e), and membranes of epithelial cells in intestinal glands were seen at a depth of 175 μm (Fig. 4f). Cells were resolved up to a depth of 250 μm (Fig. 4g and h).

## Discussion

The use of moxifloxacin, an FDA-approved antibiotic, as a cell-labeling agent for 2P imaging had been demonstrated in a previous study^8^. However, the moxifloxacin-based 2P imaging depth is shallow due to the relatively short excitation wavelength of <800 nm used. In this study, 3P excitation of moxifloxacin with a >1000 nm excitation wavelength was experimentally verified by measuring the relationship between fluorescence intensity and excitation power. 3P imaging with moxifloxacin labeling was tested in *ex vivo* mouse bladder and *ex vivo* mouse small intestine using a Ti:sapphire laser at near 1030 nm excitation wavelength, and compared with moxifloxacin-based 2P imaging using a 780 nm excitation wavelength. Although both 3P and 2P imaging visualized cellular structures via moxifloxacin labeling, moxifloxacin-based 3P imaging using longer excitation wavelengths resolved small cellular features with better contrast than moxifloxacin-based 2P imaging. In this comparison study, moxifloxacin-based 3P imaging using the Ti:sapphire oscillators could not reach the maximum imaging depth because of the low excitation energy of the lasers at longer wavelengths and the very long acquisition time. Therefore, we constructed a new 3P imaging system that uses a high-energy Yb fiber laser and used it to demonstrate *in vivo* deep tissue imaging with moxifloxacin labeling.

3P excitation typically requires higher excitation photon density and excitation sources with higher pulse energy than does 2P excitation, because the probability of 3P excitation is much lower than that of 2P excitation. This also applies to moxifloxacin-based multiphoton imaging. Moxifloxacin-based 3P imaging needs to use a laser with high pulse energy, in the range of a few tenths of a nanojoule. However, Ti:sapphire oscillators were used in this study so moxifloxacin-based 3P and 2P imaging could be compared by switching the excitation wavelength of the oscillators. In addition, 3P images were acquired by increasing the pixel dwell time of the laser scanning instead of increasing the excitation energy because the current lasers have limited energy at wavelengths >1000 nm. The maximum powers of the Ti:sapphire oscillators, i.e., 50 mW at 1000 nm and 31.5 mW at 1030 nm, were used for moxifloxacin-based 3P tissue imaging at all depths. These powers corresponded to approximately 0.5 nJ/pulse. Therefore, the pixel dwell time needed to increase rapidly as the depth increased to acquire enough fluorescence signals. The image acquisition rates for the bladder sample were 1.25 s/frame at the surface and 320 s/frame at 225 μm below the surface. Although the imaging speed at large depths was too slow for practical applications, our experiment was performed to characterize moxifloxacin-based 3P imaging and compare it with corresponding moxifloxacin-based 2P imaging.

3P excitation of moxifloxacin at >1000 nm would allow the use of commercial Yb fiber lasers, which have an 1030 nm wavelength and high pulse energies up to tenths of a microjoule per pulse, as the excitation source. Because the pulse energies of these lasers are high enough for 3P excitation of moxifloxacin, the lasers could solve the problems associated with using the Ti:sapphire oscillator for 3P imaging of biological tissues, especially at large imaging depths. We used a commercial Yb fiber amplifier for *in vivo* 3P imaging with moxifloxacin labeling of the mouse small intestine. However, the imaging speed of the *in vivo* study was not satisfactory because the Yb laser has a very low repetition rate of 102.9 kHz, 800 times lower than that of the Ti:sapphire oscillator, which runs at 80 MHz. An optimal laser for moxifloxacin-based 3P imaging would have a repetition rate of 1–2 MHz^19^ and a pulse energy of a few hundred nanojoules; such Yb fiber lasers are commercially available. Because Yb fiber lasers are currently used in high-precision ophthalmic surgeries such as laser-assisted *in situ* keratomileusis (LASIK) and cataract surgeries, moxifloxacin-based 3P imaging could be easily adapted to use similar lasers in the clinic.

Fluorescence enhancement in moxifloxacin-based 3P imaging was analyzed by conducting tissue imaging with and without the use of moxifloxacin at near 1030 nm excitation wavelength. Tissue images obtained with and without the use of moxifloxacin are shown in Supplementary Figs 1 and 2. For the imaging of the *ex vivo* mouse bladder, the autofluorescence intensities at 1000 nm excitation wavelength were negligible compared to the intensities of moxifloxacin 3P fluorescence (Supplementary Fig. 1). For the imaging of the *ex vivo* mouse small intestine, moxifloxacin 3P fluorescence was approximately 7.7 times higher than the intrinsic signals. Autofluorescent molecules excitable at 1030 nm might exist in the small intestine.

The imaging resolutions of moxifloxacin-based 3P imaging using 1030 nm excitation wavelength were similar to those of moxifloxacin-based 2P imaging using 780 nm excitation wavelength. The Rayleigh length of 3P excitation is smaller than that of 2P excitation, if the same excitation wavelength is used^20^. However, 3P excitation usually requires excitation wavelengths 1.5 times longer than those for 2P excitation for the same fluorophores. In this study, the excitation wavelength for moxifloxacin-based 3P imaging was approximately 1.3 times longer than that for the corresponding 2P imaging. Therefore, the resolutions of moxifloxacin-based 3P and 2P images were approximately the same.

The imaging depth of multiphoton microscopy is limited by the scattering of both excitation and emission photons. Although moxifloxacin-based 3P imaging reduced the effect of excitation photon scattering compared to moxifloxacin-based 2P imaging by using a long excitation wavelength, the effect of emission photon scattering remained the same. The relatively short emission wavelength of moxifloxacin is not optimal for deep tissue imaging^21,22^. However, the effect of emission photon scattering is less significant than the effect of excitation photon scattering because the non-descanned photon detector used in 2P and 3P imaging can collect scattered emission photons^23^.

The emission spectra of moxifloxacin with 3P excitation at a 1030 nm excitation wavelength and 2P excitation at a 780 nm excitation wavelength were slightly different. The emission spectrum obtained with the 1030 nm excitation wavelength had a blue-shifted emission peak when compared to the emission spectrum obtained with the 780 nm excitation wavelength. Although an emission spectrum is usually constant, independent of either the excitation wavelength or the excitation process, according to Kasha’s rule, we measured the variation in the emission spectrum with these two excitation wavelengths. This variation might result from the dependence of the emission spectrum on the different excitation wavelengths or the different nonlinear excitation processes. Because 780 nm in 2P excitation and 1030 nm in 3P excitation roughly correspond to 390 and 340 nm in one-photon (1P) excitation, different excitation wavelengths might be the cause of the variation in the emission spectrum. The phenomenon of emission spectrum variation with variation in excitation wavelength is not uncommon and has been called the “red-edge effect”^24^. For testing this phenomenon, the emission spectrum of moxifloxacin was obtained at the 2P excitation wavelengths of 700 and 780 nm, where 700 nm in 2P excitation roughly corresponds to 350 nm in 1P excitation. The emission spectrum of moxifloxacin at 700 nm 2P excitation was shifted from that at 780 nm 2P excitation and closely matched that at 1030 nm 3P excitation. To check the variation phenomenon at 1P excitation, the emission spectrum of moxifloxacin was obtained at various 1P excitation wavelengths and found to vary with the excitation wavelength. Therefore, the different emission spectra of moxifloxacin, between 780 nm in 2P excitation and 1030 nm in 3P excitation, might be due to the variation of the emission spectrum with excitation wavelength, i.e., the red-edge effect.

In this study, we demonstrated 3P imaging of biological tissues using moxifloxacin as a cell-labeling agent. Moxifloxacin fluoresced at an excitation wavelength >1000 nm as a 3P excitation process, and its excitation and emission spectra were obtained. Moxifloxacin-based 3P imaging showed better contrast and larger imaging depth than did moxifloxacin-based 2P imaging. Moxifloxacin-based 3P imaging could be useful for deep tissue imaging in both preclinical and clinical applications.

## Methods

### Multiphoton microscopy systems

A commercial and two custom-built multiphoton microscopy systems were used in this study. The commercial system (SP-5, Leica, Germany) was used to characterize moxifloxacin fluorescence at a >1000 nm excitation wavelength and to perform *ex vivo* 2P and 3P imaging of a mouse bladder. The excitation source was a Ti:sapphire femtosecond laser (Chameleon Vision, Coherent Inc., USA) with a tunable wavelength from 680 to 1080 nm, an 80 MHz repetition rate, and a 140 fs pulse width. A 40x objective lens (water immersion NA 1.1 LD C-Apochromat Corr M27, Zeiss, Germany) was used. The emitted light could be spectrally resolved using a dispersion prism and a sliding slit in the system, a setup used to obtain the moxifloxacin emission spectra. For imaging, the emitted light was spectrally collected at four detection channels by a combination of dichroic mirrors and filters. The custom-built multiphoton microscopy system was equipped with another Ti:sapphire femtosecond laser (Chameleon Ultra II, Coherent Inc., USA), and used for 2P and 3P imaging of *ex vivo* mouse small intestines. The same 40x objective lens used in the commercial system was used in the custom system. The emitted light could be separated at up to three channels using a combination of dichroic mirrors and filters. Photomultiplier tubes (H7421-40P, Hamamatsu, Japan) in photon-counting mode were used to detect the emission.

Multiphoton microscopy systems based on Ti:sapphire femtosecond lasers were used for *ex vivo* moxifloxacin-based 2P and 3P imaging of biological tissues by switching the excitation wavelength between 780 and near 1030 nm. These Ti:sapphire femtosecond laser-based multiphoton systems were not appropriate for *in vivo* moxifloxacin-based 3P imaging because the image acquisition time was too long at large depths, a result of insufficient pulse energy for efficient 3P excitation of moxifloxacin. To demonstrate *in vivo* moxifloxacin-based 3P imaging, we constructed a new 3P imaging system with a high-energy Yb fiber laser (Uranus 2500-1030-0800-PM, PolarOnyx, USA) that had a wavelength of 1034 nm, a pulse energy of ~10 µJ, and a repetition rate of 102.9 kHz. The high pulse energy, controlled electronically, allowed *in vivo* deep tissue imaging based on efficient 3P excitation of moxifloxacin. The laser beam went through a standard multiphoton microscopy system that included a beam expander (AC254-050-B and AC254-150-B, Thorlabs, USA), an *x*–*y* galvanometer scanner (GVS012/M, Thorlabs, USA), a scan lens (AC254-050-B, Thorlabs, USA) and tube lens combination (AC508-200-B, Thorlabs, USA), a dichroic mirror (T860LPXR, Chroma, USA), and an objective lens, which was the same as that used in the other multiphoton microscopy systems. A dichroic mirror (T560LPXR, Chroma, USA) and an emission filter (ET850SP, Chroma, USA) spectrally separated emitted light into two channels. Two photomultiplier tubes (R928P, Hamamatsu) detected the emitted light in photon-counting mode.

### Characterization of moxifloxacin fluorescence at a >1000 nm excitation wavelength

Ophthalmic solution (Vigamox, Alcon, Fort Worth, TX, USA) containing 5.45 mg/ml of moxifloxacin hydrochloride (pH 6.8) was used in all the experiments. The ophthalmic solution (50 μl) was placed on a microscope well glass, covered with a microscope cover glass, and sealed with nail polish. The excitation and emission spectra were measured using the commercial multiphoton microscopy system. The excitation spectrum was measured by changing the wavelength from 800 to 1050 nm in 10 nm steps and collecting the light emitted from the ophthalmic solution sample. The emission spectrum was measured from 405 to 664 nm using a 7 nm spectral step size. Once moxifloxacin fluorescence at the >1000 nm excitation wavelength was confirmed, the multiphoton excitation process was analyzed by measuring the relationship between the fluorescence intensity and the excitation power. The fluorescence intensity was measured while varying the excitation power from 8 to 18 mW at a 1030 nm excitation wavelength. The results were displayed in a log-log plot, the slope of which was estimated by least-squares curve fitting. The fluorescence intensity of moxifloxacin at a 1000 nm excitation wavelength was compared with that of DAPI (#62248, Thermo Fisher Scientific, USA), a cell nucleus-labeling dye with known multiphoton fluorescence properties^17^. Both moxifloxacin and DAPI solutions were diluted to 2.86 mM with phosphate-buffered saline.

### 2P and 3P imaging of ***ex vivo*** mouse bladder and small intestine

The SKH1-HrHr mice (5–6 weeks old) used in this study were kept under specific-pathogen-free conditions at the POSTECH Biotech Center. All procedures were performed in accordance with the relevant guidelines and regulations, and all experimental procedures performed on the animals were approved by the POSTECH Institutional Animal Care and Use Committee (approval number POSTECH-2015-0030-R2). For the *ex vivo* specimens, mice were sacrificed and the bladder or the small intestine were excised. Approximately 2.5 μl/mm^2^ of moxifloxacin ophthalmic solution was administered to the tissue, the maximum amount that the tissue could hold. The time needed for the moxifloxacin to penetrate into the tissues, i.e., the incubation time, was 20 min^8^, after which 2P and 3P imaging with moxifloxacin labeling was performed within 90 min post administration to avoid a decrease in the moxifloxacin concentration in the tissue via diffusion.

Two different excitation wavelengths, 780 and 1000 nm, were used for moxifloxacin 2P and 3P imaging of the mouse bladder, respectively. The excitation wavelength used for moxifloxacin-based 3P imaging was 1000 nm instead of 1030 nm because the bladder yielded a strong SHG signal due to collagen and current emission filters and dichroic mirrors cannot distinguish SHG from moxifloxacin fluorescence at a 1030 nm excitation wavelength. Two detection channels (Ch1: 430–480 nm and Ch2: 520–550 nm) were used and the SHG signal from collagen was omitted. The images comprised 256 × 256 pixels and the imaging field of view was 387.5 μm × 387.5 μm. The image acquisition rate for moxifloxacin-based 2P imaging was 1.25 s/frame at all imaging depths, and that for moxifloxacin-based 3P imaging varied with depth from 1.25 s/frame on the surface to 320 s/frame at 275 µm. The excitation power for 2P imaging increased with depth from 2 to 12 mW, and that for 3P imaging was 50 mW, which was the maximum power of the Ti:sapphire oscillator at the 1000 nm.

Moxifloxacin-based 2P and 3P imaging of the mouse small intestine was performed using excitation wavelengths of 780 and 1030 nm, respectively. Moxifloxacin fluorescence was collected in one channel (Ch1: 400–560 nm). The images comprised 256 × 256 pixels and the imaging field of view was 150 μm × 150 μm. The image acquisition rate for moxifloxacin-based 2P imaging was 3.3 s/frame at all imaging depths, and that for moxifloxacin-based 3P imaging varied with depth from 3.3 s/frame on the surface to 655 s/frame at 120 μm. The excitation power for 2P imaging increased with depth from 6 to 65 mW, and that for 3P imaging was 31.5 mW, which was the maximum power of the Ti:sapphire oscillator at 1030 nm.

### 3P imaging of ***in vivo*** mouse small intestine

For *in vivo* imaging, the mouse was anesthetized with a gas mixture of 1.5%/vol isoflurane (Terrell, Pirimal Critical Care, Inc., USA) and medical grade oxygen administered via face mask. The abdomen was cut open and a portion of the small intestine was gently pulled out and hung on a custom-built intestine holder (Live Cell Instrument, Korea). The intestine holder consisted of a grooved holder that kept the pulled small intestine in position. In addition, the intestine holder had a magnetically attached cover that gently pressed the small intestine. A fluid channel supplied saline to the holder groove to keep the small intestine moist. After the small intestine was administered moxifloxacin ophthalmic solution (2.5 μl/mm^2^), it was incubated for 20 min. A temperature controller kept the mouse body and pulled small intestine at 37 °C. After incubation, we performed *in vivo* moxifloxacin-based 3P imaging of the mouse small intestine using our new custom system based on the high-energy Yb fiber laser with an excitation wavelength of 1034 nm. Moxifloxacin fluorescence was collected at Ch1 (400–560 nm). The images comprised 256 × 256 pixels and the imaging field of view was 300 μm × 300 μm. The image acquisition time was 66 s/frame at all imaging depths, and the excitation power increased with depth from 5.2 to 8 mW.

### Contrast analysis of 2P and 3P tissue images

To compare moxifloxacin-based 2P and 3P imaging of biological tissue, we analyzed the variation of image contrast with depth. The image contrast was quantified by calculating the CV, which is a measure of the relative variability in the intensity of the image and is calculated as the ratio of the standard deviation to the mean. Before calculating the CV, the 2P and 3P images were preprocessed. The background of the original images was nonuniform. After removing the noise, the nonuniformity was corrected by multiplying a gain matrix the same size as the image. The gain matrix was obtained by local adaptive intensity analysis. Valid pixels containing cellular structures were selected by thresholding. The CV of an image was calculated using the valid pixels of the background-normalized 2P and 3P images. All the image-processing procedures were conducted using Matlab (R2017a, MathWorks, USA).

### Data availability

The data generated during the current study are available from the corresponding author on reasonable request.

## Electronic supplementary material

Supplementary information

## References

[1] So PT, Dong CY, Masters BR, Berland KM (2000). Two-photon excitation fluorescence microscopy. Annual review of biomedical engineering.

[2] Svoboda K, Yasuda R (2006). Principles of two-photon excitation microscopy and its applications to neuroscience. Neuron.

[3] Celli S, Albert ML, Bousso P (2011). Visualizing the innate and adaptive immune responses underlying allograft rejection by two-photon microscopy. Nature medicine.

[4] Le Dévédec SE, Lalai R, Pont C, De Bont H, Van De Water B (2011). Two-photon intravital multicolour imaging combined with inducible gene expression to distinguish metastatic behavior of breast cancer cells in vivo. Molecular Imaging and Biology.

[5] Webb RH (1996). Confocal optical microscopy. Reports on Progress in Physics.

[6] Kim B (2015). In vivo visualization of skin inflammation by optical coherence tomography and two-photon microscopy. Biomed Opt Express.

[7] Balu M (2014). Distinguishing between benign and malignant melanocytic nevi by in vivo multiphoton microscopy. Cancer research.

[8] Wang T (2016). Moxifloxacin: Clinically compatible contrast agent for multiphoton imaging. Scientific reports.

[9] Ocaña JA, Barragán FJ, Callejón M (2000). Spectrofluorimetric determination of moxifloxacin in tablets, human urine and serum. Analyst.

[10] Robertson SM (2005). Ocular pharmacokinetics of moxifloxacin after topical treatment of animals and humans. Survey of ophthalmology.

[11] Hell SW (1996). Three-photon excitation in fluorescence microscopy. Journal of Biomedical Optics.

[12] Yildirim M, Durr N, Ben-Yakar A (2015). Tripling the maximum imaging depth with third-harmonic generation microscopy. Journal of biomedical optics.

[13] Norris G, Amor R, Dempster J, Amos WB, McConnell G (2012). A promising new wavelength region for three‐photon fluorescence microscopy of live cells. Journal of microscopy.

[14] Huland DM (2013). Three-photon excited fluorescence imaging of unstained tissue using a GRIN lens endoscope. Biomedical optics express.

[15] Horton NG (2013). In vivo three-photon microscopy of subcortical structures within an intact mouse brain. Nature photonics.

[16] Lee, S. *et al*. *In vivo* 3D measurement of moxifloxacin and gatifloxacin distributions in the mouse cornea using multiphoton microscopy. *Scientific reports* **6** (2016). 10.1038/srep25339 PMC4853790 27138688

[17] Xu C, Williams R, Zipfel W, Webb WW (1996). Multiphoton excitation cross-sections of molecular fluorophores. Bioimaging.

[18] Frith, C. H., Townsend, J. W. & Ayres, P. H. in Urinary System 281–284 (Springer, 1986).

[19] Cheng L-C, Horton NG, Wang K, Chen S-J, Xu C (2014). Measurements of multiphoton action cross sections for multiphoton microscopy. Biomedical optics express.

[20] Shi L, Rodríguez-Contreras A, Alfano RR (2014). Gaussian beam in two-photon fluorescence imaging of rat brain microvessel. Journal of biomedical optics.

[21] Sordillo LA, Pu Y, Pratavieira S, Budansky Y, Alfano RR (2014). Deep optical imaging of tissue using the second and third near-infrared spectral windows. Journal of biomedical optics.

[22] Shi L, Sordillo LA, Rodríguez‐Contreras A, Alfano R (2016). Transmission in near‐infrared optical windows for deep brain imaging. Journal of biophotonics.

[23] Dunn AK, Wallace VP, Coleno M, Berns MW, Tromberg BJ (2000). Influence of optical properties on two-photon fluorescence imaging in turbid samples. Applied optics.

[24] Demchenko AP (2002). The red‐edge effects: 30 years of exploration. Luminescence.

