# Supplementary material for: Three-photon tissue imaging using moxifloxacin
Source: Sci Rep. 2018 Jun 20;8:9415. doi: 10.1038/s41598-018-27371-8 (PMC6010410; doi:10.1038/s41598-018-27371-8)

Three-photon tissue imaging by using moxifloxacin

Seunghun Lee[1], Jun Ho Lee[1], Taejun Wang[2], Won Hyuk Jang[2], Yeoreum Yoon[1], Bumju Kim[2], Yong Woong Jun[3], Myoung Joon Kim[4] & Ki Hean Kim[1,2,*]

[1] Department of Mechanical Engineering, Pohang University of Science and Technology, 77 Cheongam-ro, Nam-gu, Pohang, Gyeongbuk, 37673, Rep. of Korea

[2] Division of Integrative Biosciences and Biotechnology, Pohang University of Science and Technology, 77 Cheongam-ro, Nam-gu, Pohang, Gyeongbuk, 37673, Rep. of Korea

[3] Department of Chemistry, Pohang University of Science and Technology, 77 Cheongam-ro, Nam-gu, Pohang, Gyeongbuk, 37673, Rep. of Korea

[4] Department of Ophthalmology, University of Ulsan College of Medicine, Asan Medical Center, 88 Olympic-ro 43-gil, Songpa-gu, Seoul, 05505, Rep. of Korea

* Corresponding author: kiheankim@postech.ac.kr, Tel:+82 54 279 2190, Fax: +82 54 279 5899

Table of contents

Supplementary figure S1 – Moxifloxacin based three-photon (3P) images and intrinsic fluorescence based multi-photon images of an ex vivo mouse bladder at 1000 nm excitation wavelength.

Supplementary figure S2 – Moxifloxacin based 3P images and intrinsic fluorescence based multi-photon images of an ex vivo mouse small intestine at 1030 nm excitation wavelength.

Supplementary figure legends

Supplementary figure S1 – Moxifloxacin based 3P images and intrinsic fluorescence based multi-photon images of an ex vivo mouse bladder at 1000 nm excitation wavelength. (a – d) : Moxifloxacin based 3P images at depths of 0, 25, 50 and 100 μm from the laminal side. (e – h) : Intrinsic fluorescence based multi-photon images at the depths of 0, 25, 50 and 100 μm from the laminal side. Scale bar = 50 μm.

Supplementary figure S2– Moxifloxacin based 3P images and intrinsic fluorescence based multi-photon images of an ex vivo mouse small intestine at 1030 nm excitation wavelength. (a – d) : Moxifloxacin based 3P images at depths of 0, 25, 50 and 75 μm from the serosa. (e – h) : Intrinsic fluorescence based multi-photon images at the depths of 0, 25, 50 and 75 μm from the serosa. Scale bar = 50 μm.

Supplementary figures

Supplementary figure S1

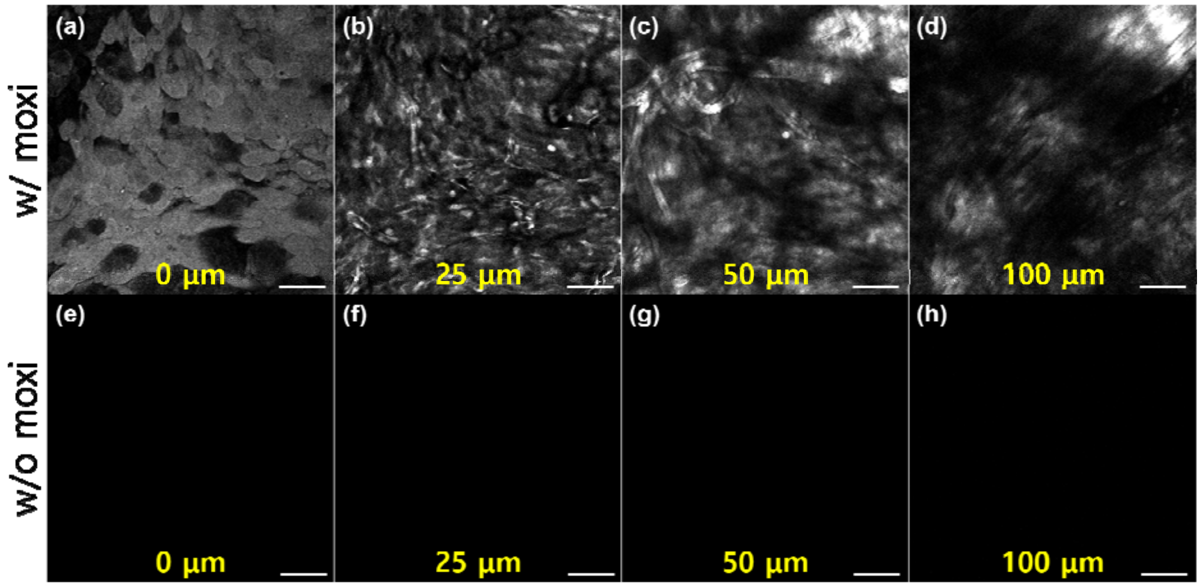

Supplementary figure S2

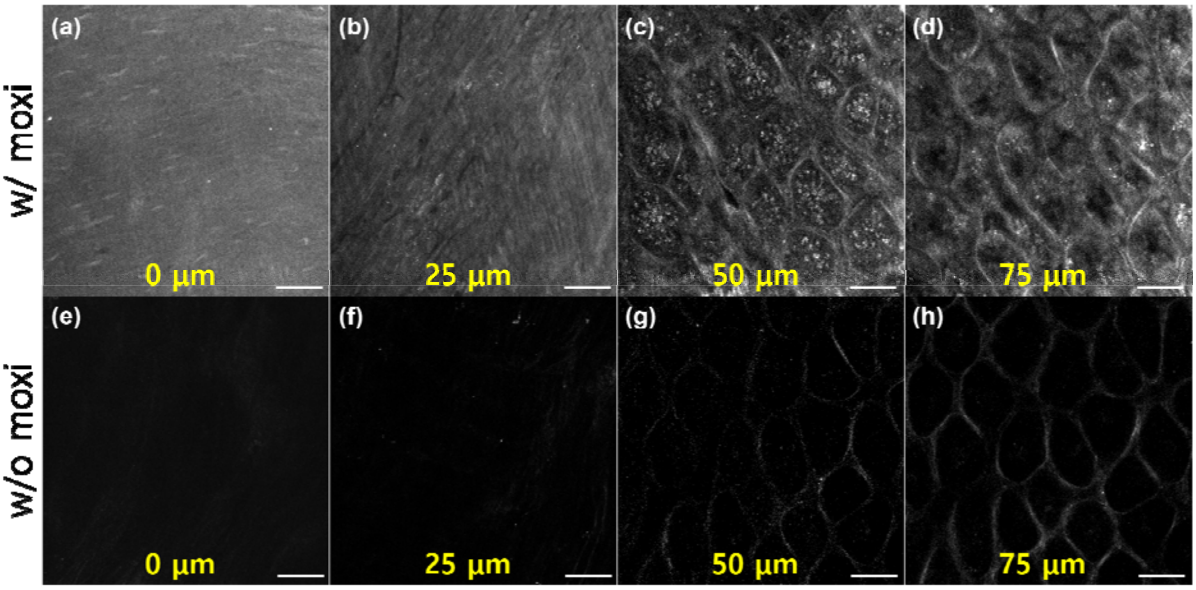

Supplement: Supplementary file 1 — Supplementary information [file 41598_2018_27371_MOESM1_ESM.pdf]
